# U.S. Public Opinion About Immigration Enforcement in Sensitive Locations

**DOI:** 10.1007/s10903-025-01772-0

**Published:** 2025-09-01

**Authors:** Christine Crudo Blackburn, Timothy Callaghan

**Affiliations:** 1 Texas A&M University, College Station, USA; 2 Boston University, Boston, USA

**Keywords:** Immigration enforcement, Healthcare access, Immigrants, Survey, Immigration policy

## Abstract

In 2011, the Director of U.S. Immigration and Customs Enforcement (ICE) issued a memorandum titled, “Enforcement Actions at or Focused on Sensitive Locations.” The memorandum stated that immigration enforcement actions should not occur at specified sensitive locations, which included schools, healthcare facilities, places of worship, sites of public religious ceremony, and sites of public demonstration. These policies were rescinded in January 2025, creating the possibility of enforcement actions in these locations. The aim of this study was to investigate U.S. public opinion regarding immigration enforcement in sensitive locations. We conducted an online survey of 3,563 American adults with quotas implemented on gender, age, race, and geographic location. The survey ran from January 23 to February 3, 2025. 3,563 individuals completed the survey. Age and political identification were the most consistent predictors of support for rescinding sensitive locations policy, as well as in the belief that such enforcement would not deter care seeking. Sympathy towards immigrants was also a predictor of support for sensitive locations policy. Our findings suggest that the majority of Americans do not think that ICE’s sensitive locations policy should be rescinded and believe that rescinding this policy will deter undocumented immigrants from seeking needed medical care for themselves and their children. A lack of support for rescinding this policy suggests that it should be reinstated, a move that would help to protect healthcare access for undocumented people and their families.

**Supplementary Information:**

The online version contains supplementary material available at 10.1007/s10903-025-01772-0.

## Introduction

In 2011, the Director of U.S. Immigration and Customs Enforcement (ICE) issued a memorandum titled, “Enforcement Actions at or Focused on Sensitive Locations [1].” The memorandum stated that immigration enforcement actions should not occur at specified sensitive locations, which included schools, healthcare facilities, places of worship, sites of public religious ceremony, and sites of public demonstration [1]. It outlined the types of actions that ICE could not take place in these locations, including arrests, interviews, searches, and surveillance [1]. In 2021, an updated version was issued, which expanded the protection offered by the sensitive locations policy [2, 3]. Then, on January 20, 2025, President Trump signed an executive order that rescinded the sensitive locations policy [4]. The rescinding of ICE’s sensitive locations policy has created fear among immigrants and a scramble to understand the implications of this policy change at sites previously designated as sensitive locations [5, 6]. There is reason to believe that it could lead to a reduction in healthcare access for the estimated 11 million undocumented immigrants in the United States, even in emergency situations.

In the United States, the Emergency Medical Treatment and Labor Act (EMTALA) requires that individuals be provided with emergency medical care regardless of their immigration status or ability to pay, but immigration policies and anti-immigrant rhetoric can serve as barriers to accessing both routine healthcare and emergency care [7–9]. Fear of encountering immigration officers in healthcare settings raises concern among undocumented immigrants about accessing care or about visiting family members in the hospital [10]. Fear of deportation is one of the primary reasons that undocumented individuals avoid care during times of aggressive immigration enforcement or high anti-immigrant sentiment [11–13]. The fear created by immigration policies and enforcement can affect not only healthcare access among undocumented people, but also among legal immigrants [10].

Importantly, there is agreement in the literature that a direct relationship exists between anti-immigration policies and access to health services [14]. Previous research demonstrates that following proposed public charge changes in 2017, there was a significant decrease in well-child visits among children of immigrants compared to children of U.S. born mothers [15], and this chilling effect in healthcare utilization became even more pronounced following the 2019 implementation of those changes [16]. This suggests that rescinding the sensitive locations policy could have a substantial, negative impact on healthcare access for undocumented immigrants, legal immigrants, and U.S. citizens in mixed status families. Healthcare provision is made more difficult as undocumented parents and patients are wary of divulging too much information that could be used against them and lead to deportation, leading some to delay seeking care until the problem has become severe [17–19]. Immigration enforcement also has a negative mental health impact, creating trauma, stress, and anxiety [18, 20, 21]. Lastly, it is important to note that previous research has suggested that the impacts of immigration enforcement do not just affect the health and well-being of the undocumented individual, but also that U.S. citizen children from mixed status families have worse physical outcomes than children from families in which all members have legal status in the United States [22].

In addition to these well-documented direct impacts on healthcare access, health-seeking behavior, and health outcomes within the healthcare context, immigration enforcement in other sensitive locations such as schools and churches, can have additional indirect negative impacts on health outcomes for immigrant children or children from mixed status families. Previous research has found that the threat of immigration enforcement affects student learning in schools with many immigrant children expressing overt fear of immigration enforcement and teachers noting increased emotional and behavioral problems among immigrant students [23]. In addition, interior immigration enforcement has been shown to increase chronic absenteeism among immigrant students [24] and self-reported feelings of sadness [25]. Importantly, previous research has also shown that immigration raids have lasting, negative impacts on immigrant children, which include increased substance use disorder, depression, suicide attempts, and self-harm [26]. The previous research is clear that when interior immigration enforcement (i.e., immigration enforcement within the United States rather than along the border or at ports of entry) increases, immigrants and their children experience increased fear of deportation, which has negative effects on their physical and mental health [7–9, 26, 27].

The role that immigration enforcement plays in healthcare avoidance and the impact that it has on the health outcomes of immigrants and their families is well documented [10, 13, 14, 17, 21, 28]. Rescinding the sensitive locations policy could increase the rate of healthcare avoidance and healthcare delay among undocumented immigrants and their family members, which makes investigating public support for removing these protections vital to future decision-making. Policy decisions that could negatively impact the population and its health must be assessed to examine whether they are, in fact, supported by the public. Considering recent changes to the sensitive locations policy, we conducted a national survey to investigate U.S. public opinion regarding immigration enforcement in sensitive locations and beliefs about effects on healthcare access for undocumented people and their families when these policies are rescinded.

## Methods

### Data Collection

We developed an original survey which was programmed in Qualtrics and administered by the survey research firm Cint Theorem, formerly Lucid, which draws quota samples from its large, opt-in panel of potential survey respondents. Through the implementation of quotas for race/ethnicity, gender, age, and geographic region, Cint Theorem produces samples that are demographically representative of the US population. Critically, research consistently demonstrates the high-quality nature of the samples produced by Cint, as illustrated through a wide body of social and health sciences research [29–33]. Survey respondents were compensated for their participation by Cint USA. The survey ran from January 23 to February 3, 2025. During that time 4,061 U.S. adults initiated the survey and 3,563 completed it, for a completion rate of 85%.

### Data Analysis

We examined public awareness of sensitive locations policies, beliefs about the involvement of immigration politics in healthcare, agreement with immigration enforcement in sensitive locations, and belief about whether such enforcement would deter care-seeking. For the first two outcome measures, we asked respondents to select their level of agreement with survey questions that stated (1) “I am aware the Immigration and Customs Enforcement (ICE) maintains a policy prohibiting immigration enforcement activities in sensitive locations such as hospitals, schools, and churches,” and (2) “Politics related to immigration have no place in the U.S. healthcare system.” Response choices included “strongly agree,” “somewhat agree,” “neither agree nor disagree,” “somewhat disagree,” and “strongly disagree.”

Next, the survey asked about respondents’ agreement with enforcement in sensitive locations, stating (1) “Immigration enforcement should not occur in hospitals,” (2) “Immigration enforcement should not occur in schools,” and (3) “Immigration enforcement should not occur in churches.” Again, respondents were provided with five response choices from strongly agree to strongly disagree.

Lastly, respondents were asked about their level of agreement related to the possible impact of immigration enforcement in sensitive locations with the following questions, (1) “Immigration enforcement in hospitals will deter undocumented immigrants from seeking needed medical care,” (2) “Immigration enforcement in hospitals will deter undocumented parents of U.S. citizen children (0–18 years of age) from taking these children to get needed medical care,” and (3) “Immigration enforcement in hospitals will deter undocumented immigrants from calling 911 in an emergency.” Response choices presented the same five levels of agreement for respondents to choose from.

In addition to questions related to sensitive locations policy awareness, support, and perceived effects which serve as outcome measures in our analysis, our survey contained questions which serve as explanatory measures in our analysis, including self-identified gender (male, female, trans male/man, trans female/women, genderqueer/gender non-conforming, other), age (18–24 years old, 25–34 years old, 35–44 years old, 45–54 years old, 55–64 years old, 65–74 years old, 75+), race/ethnicity (American Indian or Alaska Native, Asian, Black or African American, Native Hawaiian or Pacific Islander, White, Hispanic or Latino, Two or more races, other), level of educational attainment (less than high school, high school diploma or equivalent, some college no degree, technical certification/degree, associate’s degree, bachelor’s degree, master’s degree, doctoral or professional degree), employment status (employed full time, employed part time, unemployed looking for work, unemployed not looking for work, retired, stay-at-home caregiver for family member, full-time student), household income (less than $25,000, $25,000 to $49,999, $50,000 to $74,999, $75,000 to $99,999, $100,000 to $149,999, $150,000 or more), rurality (rural, suburban, urban), political identity (Democrat, Independent, Republican), geographic location (select their state of residence from a drop down menu), military experience (active duty military, reserve military, National Guard, veteran, cadet or undergoing military education, no military experience), and sympathy towards immigrants (very sympathetic, somewhat sympathetic, somewhat unsympathetic, very unsympathetic).

Given the ordinal nature of our outcome measures, our analysis in this manuscript relied on ordinal logistic regression. Importantly however, in the supplemental materials for this manuscript, we present alternative model specifications relying on ordinary least squares regression with each outcome measure kept on its 5-point scale as well as multivariable logistic regression models, where each outcome measure was recategorized as a binary measure, with the outcome coded as a 1 if respondents agreed strongly or somewhat with a given question and coded as 0 otherwise.

We additionally characterized the study population using descriptive statistical analysis and we conducted univariate regression analysis for all dependent variables with all independent variables. This analysis can also be found in the supplemental materials. We set the threshold for statistical significance at an alpha value of 0.05 (*p* <.05). All analysis was conducted in February 2025 using StataMP 18.5.

### Ethics Statement

Our study was declared exempt by the [institution name redacted for review purposes]. Respondents were presented with an electronic consent form upon accessing the survey. At the end of the consent document, respondents who agreed to participate were allowed to continue with the survey. Those who selected, “I disagree,” were exited from the survey.

## Results

In total, 3,563 individuals completed the survey. The majority of participants were female (51.48%), White (62.22%), had less than a bachelor’s degree level of education (72.83%), and had a household income of less than $75,000 (72.91%). While participants were almost evenly split in political identity between Democrat (34.13%), Independent (37.22%), and Republican (28.65%), the largest number of participants came from suburban areas (45.92%).

Our analysis found that the majority of participants agreed that immigration enforcement should not take place in hospitals (59.82%), schools (58.73%), and churches (60.13%). Additionally, the majority of study participants responded that they believed conducting immigration enforcement in hospitals would deter undocumented immigrants from seeking needed medical care (65.98%), would deter undocumented parents from taking their U.S. citizen children to get needed medical care (64.61%), and would deter undocumented immigrants from calling 9–11 when they needed emergency care (64.18%). These and additional descriptive statistics are available in Table 1.

**Table 1:** Demographic and socioeconomic characteristics of survey respondents (*n*=3,563)

Variable	*n* (%)
Gender	
*Male*	1,796 (51.48)
*Female*	1,693 (48.52)
Age	
*18-24 years*	436 (12.34)
*25-34 years*	689 (19.50)
*35-44 years*	634 (17.95)
*45-54 years*	553 (15.65)
*55-64 years*	509 (14.41)
*≥ 65 years*	712 (20.15)
Race	
*White*	2,200 (62.22)
*Asian*	199 (5.63)
*Black or African American*	489 (13.83)
*Hispanic or Latino*	482 (13.63)
*Other*	166 (5.63)
Education	
*High school or less*	1,207 (34.15)
*Some college*	832 (23.54)
*Tech or associate degree*	536 (15.17)
*Bachelor’s degree*	642 (18.17)
*Graduate degree*	317 (8.97)
Employment Status	
*Employed*	1,937 (54.81)
*Unemployed*	1,597 (45.19)
Household Income (US $)	
*< $25*,*000*	864 (24.58)
*$25*,*000–$49*,*999*	945 (26.88)
*$50*,*000–$74*,*999*	754 (21.45)
*$75*,*000–$99*,*999*	402 (11.44)
*$100*,*000-$149*,*000*	353 (10.04)
*$150*,*000 or more*	197 (5.60)
Rurality	
*Rural*	928 (26.60)
*Suburban*	1,602 (45.92)
*Urban*	959 (27.49)
Political Identity	
*Democrat*	1,202 (34.13)
*Independent*	1,009 (28.65)
*Republican*	1,311 (37.22)
US Census Region	
*Northeast*	696 (21.16)
*Midwest*	725 (22.04)
*West*	728 (22.13)
*South*	1,141 (34.68)
Military Experience	
*Yes*	490 (13.89)
*No*	3,038 (86.11)
Sympathy toward Immigrants	
*Unsympathetic*	1,625 (46.02)
*Sympathetic*	1,906 (53.98)
Enforcement in Hospitals	
*Yes*	671 (19.00)
*Unsure*	748 (21.18)
*No*	2,113 (59.82)
Enforcement in Schools	
*Yes*	742 (21.00)
*Unsure*	716 (20.27)
*No*	2,075 (58.73)
Enforcement in Churches	
*Yes*	667 (18.93)
*Unsure*	738 (20.94)
*No*	2,119 (60.13)
Deter Care-Seeking (Adults)	
*Yes*	2,329 (65.98)
*Unsure*	839 (23.77)
*No*	362 (10.25)
Deter Care-Seeking (Children)	
*Yes*	2,282 (64.61)
*Unsure*	854 (24.18)
*No*	396 (11.21)
Deter Care-Seeking (911)	
*Yes*	2,262 (64.18)
*Unsure*	862 (24.45)
*No*	401 (11.38)

In univariate models available in the supplemental materials, we found statistically significant relationships between gender, age, race, education, employment status, rurality, political identity, and sympathy towards immigrants and measures related to awareness of sensitive locations policy and agreement with the statement that immigration politics has no place in the U.S. healthcare system. Additionally, statistically significant relationships were demonstrated between gender, age, race, education, income, political identity, and sympathy towards immigrants and measures of agreement with the violation of sensitive locations policy in hospitals, schools, and churches. Lastly, univariate models demonstrated a statistically significant relationship between race, education, income, political identity, geographic region, and sympathy towards immigrants and measures related to agreement that immigration enforcement in hospitals would deter care-seeking among undocumented immigrants.

Ordinal logistic regression models examining awareness of the sensitive locations policy and the belief that immigration politics do not belong in the U.S. healthcare system suggested that older respondents and respondents who identified as Independents were less likely to be aware that ICE maintained a sensitive locations policy (Table 2). Additionally, those with some college education but no degree and those with a technical certificate/degree or associate degree were 20% and 21% less likely, respectively, to be aware that ICE maintains sensitive locations policy compared to those with a high school education or less.

**Table 2:** Characteristics associated with awareness of sensitive locations policy and belief that immigration politics do not belong in the U.S. Healthcare system (*N*=3,176)

Characteristic	Awareness	Immigration Politics in Healthcare
Gender		
*Female*	*ref*	*ref*
*Male*	1.07 (0.07)	1.06 (0.07)
	[0.94 - 1.22]	[0.93 - 1.21]
Age		
*18-24 years*	*ref*	*ref*
*25-34 years*	0.91 (0.11)	0.78* (0.09)
	[0.72 - 1.15]	[0.61 - 0.99]
*35-44 years*	0.84 (0.10)	0.82 (0.10)
	[0.66 - 1.07]	[0.64 - 1.04]
*45-54 years*	0.67** (0.09)	0.69** (0.09)
	[0.53 - 0.86]	[0.53 - 0.88]
*55-64 years*	0.57** (0.07)	0.64** (0.08)
	[0.44 - 0.73]	[0.50 - 0.83]
*≥ 65 years*	0.60** (0.08)	0.71** (0.09)
	[0.46 - 0.78]	[0.55 - 0.92]
Race		
*White*	*ref*	*ref*
*Black or African American*	1.10 (0.12)	0.98 (0.11)
	[0.89 - 1.36]	[0.79 - 1.21]
*Hispanic or Latino*	1.03 (0.11)	1.01 (0.10)
	[0.84 - 1.26]	[0.82 - 1.23]
*Asian*	0.90 (0.13)	0.82 (0.12)
	[0.68 - 1.19]	[0.63 - 1.23]
*Other*	0.96 (0.16)	0.89 (0.15)
	[0.70 - 1.34]	[0.64 - 1.23]
Education		
*High school or less*	*ref*	*ref*
*Some college*	0.80** (0.07)	0.85 (0.08)
	[0.67 - 0.95]	[0.71 - 1.01]
*Tech or associate degree*	0.79* (0.08)	0.91 (0.09)
	[0.64 - 0.96]	[0.74 - 1.11]
*Bachelor’s degree*	0.96 (0.10)	0.89 (0.09)
	[0.78 - 1.17]	[0.73 - 1.09]
*Graduate degree*	1.00 (0.13)	1.14 (0.15)
	[0.77 - 1.29]	[0.88 - 1.49]
Employment Status		
*Employed*	*ref*	*ref*
*Unemployed*	0.94 (0.07)	0.88 (0.07)
	[0.80 - 1.09]	[0.75 - 1.02]
Household Income (US $)		
*< $25*,*000*	*ref*	*ref*
*$25*,*000–$49*,*999*	1.10 (0.10)	1.09 (0.10)
	[0.92 - 1.32]	[0.91- 1.31]
*$50*,*000–$74*,*999*	1.17 (0.11)	1.01 (0.10)
	[0.88 - 1.30]	[0.83 - 1.24]
*$75*,*000–$99*,*999*	0.98 (0.12)	0.97 (0.12)
	[0.77 - 1.25]	[0.76 - 1.24]
*$100*,*000-$149*,*000*	1.02 (0.14)	1.07 (0.14)
	[0.79 - 1.33]	[0.82 - 1.39]
*$150*,*000 or more*	1.19 (0.19)	0.96 (0.16)
	[0.87 - 1.63]	[0.69 - 1.33]
Rurality		
*Rural*	*ref*	*ref*
*Suburban*	1.09 (0.09)	0.86 (0.07)
	[0.93 - 1.27]	[0.74 - 1.01]
*Urban*	1.15 (0.11)	0.96 (0.09)
	[0.96 - 1.38]	[0.80 - 1.14]
Political Identity		
*Democrat*	*ref*	*ref*
*Independent*	0.58** (0.05)	0.49** (0.04)
	[0.49 - 0.69]	[0.42 - 0.58]
*Republican*	0.85 (0.08)	0.43** (0.04)
	[0.73 - 1.01]	[0.37 - 0.51]
US Census Region		
*Northeast*	*ref*	*ref*
*Midwest*	1.08 (0.10)	1.17 (0.11)
	[0.90 - 1.30]	[0.96 - 1.42]
*West*	1.08 (0.11)	1.30** (0.13)
	[0.90 - 1.31]	[1.06 - 1.58]
*South*	1.04 (0.09)	1.16 (0.10)
	[0.87 - 1.23]	[0.97 - 1.38]
Military Status		
*No military experience*	*ref*	*ref*
*Military Experience*	1.20 (0.12)	1.12 (0.12)
	[0.98 - 1.46]	[0.92 - 1.37]
Sympathy		
*Unsympathetic*	*ref*	*ref*
*Sympathetic*	1.32** (0.09)	2.64** (0.18)
	[1.15 - 1.51]	[2.31 - 3.03]

Cell entries represent results of ordinal logistic regression models using odds ratios with standard errors in parentheses and confidence intervals in brackets. From left to right, the dependent variables for the two models indicate respondent agreement that (1) they are aware that ICE maintains sensitive locations policy, and (2) belief that politics related to immigration have no place in the U.S. healthcare system. Dependent variables for the models are Likert scales ranging from “strongly disagree” (1) to “strongly agree” (5)

* *p*<.05 ** *p*<.01

Independents were 51% less likely and Republicans were 57% less likely to agree that immigration politics had no place in the U.S. healthcare system compared to Democrats. Conversely, respondents who noted that they were geographically located in the West were 30% more likely and those who expressed sympathy towards immigrants were 164% more likely to agree that immigration politics has no place in the U.S. healthcare system compared to those who resided in the Northeast and those who expressed that they were unsympathetic towards immigrants, respectively. Those who expressed sympathy towards immigrants were 32% more likely than those who did not, to say that they were aware of sensitive locations policy (Table 2).

Ordinal logistic regression models investigating agreement with immigration enforcement actions in sensitive locations, specifically hospitals, schools, and churches (Table 3), suggest that older respondents and those who identified as a Republican or Independent was associated with higher levels of agreement that immigration enforcement should take place in all three of these locations. In contrast, respondents with a graduate degree were more likely to agree that immigration enforcement should not occur in these three locations compared to those with a high school education or less. Race and ethnicity were statistically significant as well, with respondents who identified as Hispanic or Latino being 25% less likely to agree that immigration enforcement should occur in churches, Black or African American respondents 21% more likely to agree immigration enforcement should take place in hospitals, and Asian respondents 26% more likely to agree that immigration enforcement should take place in schools and 25% more likely to agree that immigration enforcement should take place in churches compared to respondents who identified as White. Additionally, respondents with a household income of $100,000-$149,999 were 31% less likely to agree that immigration enforcement should occur in schools compared to those with a household income of less than $25,000. Respondents who expressed sympathy toward immigrants were 294%, 317%, and 229% more likely than those who were unsympathetic toward immigrants to believe that immigration enforcement should not take place in hospitals, schools, and churches, respectively.

**Table 3:** Characteristics associated with agreement with immigration enforcement actions in sensitive locations (*N*=3,174)

Characteristic	Hospitals	Schools	Churches
Gender			
*Female*	*ref*	*ref*	*ref*
*Male*	0.86* (0.06)	0.83** (0.06)	0.89 (0.06)
	[0.75 - 0.99]	[0.73 - 0.96]	[0.78 −1.03]
Age			
*18-24 years*	*ref*	*ref*	*ref*
*25-34 years*	0.88 (0.11)	0.89 (0.11)	0.88 (0.11)
	[0.69 - 1.12]	[0.69 - 1.14]	[0.68 - 1.12]
*35-44 years*	0.81 (0.10)	0.93 (0.12)	0.83 (0.11)
	[0.63 - 1.04]	[0.72 - 1.19]	[0.65 - 1.07]
*45-54 years*	0.65** (0.09)	0.65** (0.09)	0.66** (0.09)
	[0.50 - 0.84]	[0.50 - 0.84]	[0.51 - 0.86]
*55-64 years*	0.66** (0.09)	0.59** (0.08)	0.64** (0.09)
	[0.51 - 0.86]	[0.45 - 0.78]	[0.49 - 0.84]
*≥ 65 years*	0.62** (0.09)	0.60** (0.08)	0.70** (0.10)
	[0.47 - 0.81]	[0.45 - 0.78]	[0.54 - 0.93]
Race			
*White*	*ref*	*ref*	*ref*
*Black or African American*	0.79* (0.09)	0.86 (0.10)	0.84 (0.09)
	[0.64 - 0.98]	[0.69 - 1.07]	[0.68 - 1.04]
*Hispanic or Latino*	1.17 (0.13)	1.23 (0.13)	1.25* (0.14)
	[0.95 - 1.45]	[0.90 - 1.53]	[1.01 - 1.55]
*Asian*	0.79 (0.12)	0.74* (0.11)	0.75 (0.11)
	[0.59 - 1.05]	[0.56 - 0.99]	[0.56 - 1.00]
*Other*	0.78 (0.13)	1.01 (0.17)	0.89 (0.15)
	[0.59 - 1.09]	[0.72 - 1.41]	[0.64 - 1.24]
Education			
*High school or less*	*ref*	*ref*	*ref*
*Some college*	0.98 (0.09)	1.01 (0.09)	1.01 (0.09)
	[0.82 - 1.17]	[0.84 −1.21]	[0.84 - 1.20]
*Tech or associate degree*	0.88 (0.09)	0.79* (0.08)	0.90 (0.09)
	[0.72 - 1.09]	[0.65-0.98]	[0.73 −1.11]
*Bachelor’s degree*	1.07 (0.11)	1.04 (0.11)	1.10 (0.12)
	[0.87 - 1.32]	[0.84 - 1.28]	[0.89 - 1.36]
*Graduate degree*	1.36* (0.19)	1.33* (0.19)	1.40* (0.20)
	[1.04 - 1.80]	[1.01 - 1.76]	[1.06 - 1.85]
Employment Status			
*Employed*	*ref*	*ref*	*ref*
*Unemployed*	0.93 (0.07)	0.91 (0.07)	1.00 (0.08)
	[0.79 - 1.08]	[0.78 - 1.07]	[0.85 - 1.17]
Household Income (US $)			
*< $25*,*000*	*ref*	*ref*	*ref*
*$25*,*000–$49*,*999*	1.06 (0.10)	1.15 (0.11)	1.09 (0.10)
	[0.84 - 1.22]	[0.95 - 1.38]	[0.90 - 1.31]
*$50*,*000–$74*,*999*	0.92 (0.10)	1.17 (0.12)	1.05 (0.11)
	[0.75 - 1.13]	[0.95 - 1.43]	[0.85 - 1.29]
*$75*,*000–$99*,*999*	0.99 (0.13)	0.99 (0.13)	0.82 (0.10)
	[0.77 - 1.27]	[0.77 - 1.28]	[0.64 - 1.05]
*$100*,*000-$149*,*999*	1.11 (0.16)	1.32* (0.18)	1.06 (0.15)
	[0.95 - 1.47]	[1.00 - 1.74]	[0.81 - 1.39]
*$150*,*000 or more*	0.95 (0.16)	1.08 (0.18)	0.90 (0.15)
	[0.68 - 1.32]	[0.78 - 1.51]	[0.64 - 1.25]
Rurality			
*Rural*	*ref*	*ref*	*ref*
*Suburban*	1.08 (0.09)	1.03 (0.08)	1.01 (0.08)
	[0.92 - 1.27]	[0.88 - 1.21]	[0.86 - 1.19]
*Urban*	1.17 (0.11)	1.14 (0.11)	1.06 (0.10)
	[0.97 - 1.41]	[0.95 - 1.37]	[0.88 - 1.27]
Political Identity			
*Democrat*	*ref*	*ref*	*ref*
*Independent*	0.52** (0.05)	0.47** (0.04)	0.50** (0.04)
	[0.43 - 0.61]	[0.39 - 0.55]	[0.42 - 0.59]
*Republican*	0.37** (0.03)	0.33** (0.03)	0.35** (0.03)
	[0.31 - 0.44]	[0.27 - 0.39]	[0.30 - 0.42]
US Census Region			
*Northeast*	*ref*	*ref*	*ref*
*Midwest*	1.09 (0.11)	1.03 (0.10)	0.93 (0.09)
	[0.90 - 1.33]	[0.85 - 1.26]	[0.77 - 1.14]
*West*	1.10 (0.11)	1.02 (0.11)	1.06 (0.11)
	[0.90 - 1.35]	[0.84 - 1.26]	[0.87 - 1.30]
*South*	1.12 (0.10)	1.04 (0.10)	1.04 (0.10)
	[0.94 - 1.34]	[0.87 - 1.25]	[0.87 - 1.25]
Military Status			
*No military experience*	*ref*	*ref*	*ref*
*Military Experience*	1.12 (0.12)	1.11 (0.12)	1.14 (0.12)
	[0.91 −1.37]	[0.91 - 1.37]	[0.93 - 1.41]
Sympathetic			
*Unsympathetic*	*ref*	*ref*	*ref*
*Sympathetic*	3.94** (0.28)	4.17** (0.30)	3.29** (0.23)
	[3.42 - 4.54]	[3.62 - 4.81]	[2.86 - 3.79]

Cell entries represent results of ordinal logistic regression models using odds ratios with standard errors in parentheses and confidence intervals in brackets. From left to right, the dependent variables for the three models indicate that the respondent agrees that (1) immigration enforcement should NOT occur in hospitals, (2) immigration enforcement should NOT occur in schools, and (3) immigration enforcement should NOT occur in churches. Dependent variables for the models are Likert scales ranging from “strongly disagree” (1) to “strongly agree” (5)

* *p*<.05 ** *p*<.01

Ordinal logistic regression models examining the perceived effects of immigration enforcement in hospitals (Table 4) suggests that respondents who identified as Independents were 46% less likely and those who identified as Republicans were 55% less likely to believe immigration enforcement in hospitals would deter undocumented parents of U.S. citizen children from bringing them in for needed medical care compared to respondents who identified as Democrat, respectively. Those who identified as Independent or Republican, also were 42% and 47% less likely to agree that rescinding sensitive locations policy would deter undocumented adults from seeking care or calling 911 compared to respondents who identified as Democrats. In contrast, respondents with household incomes above $25,000 were more likely to agree that immigration enforcement in hospitals would deter undocumented immigrants from seeking care or calling 911 compared to those with household incomes of less than $25,000. Black or African American respondents were 34% less likely to believe that immigration enforcement would deter undocumented immigrants from seeking needed medical care and 25% less likely to agree that it would deter undocumented immigrants from calling 9–11 and Asian respondents were 31% less likely to believe that enforcement would deter undocumented immigrants from calling 911 for needed emergency care compared to respondents who identified as White. Respondents who identified as Hispanic or Latino were 19% less likely to agree that rescinding sensitive locations policy would deter needed medical care for U.S. citizen children of undocumented parents compared to respondents who identified as White. Those who expressed sympathy for immigrants were 133% more likely agree that it would deter care for undocumented individuals, 147% more likely to agree it would deter undocumented parents from seeking care for their U.S. citizen children, and 128% more likely to agree that it would deter undocumented immigrants from calling 9–11 compared to those why said they were unsympathetic towards immigrants.

**Table 4:** Characteristics associated with agreement regarding the effects of immigration enforcement in hospitals (*N*=3,172)

Characteristic	Deter Care-Seeking	Deter Care-Seeking for Minors	Deter 9-11 Calls
Gender			
*Female*	*ref*	*ref*	*ref*
*Male*	0.99 (0.07)	0.94 (0.06)	1.00 (0.07)
	[0.86 - 1.13]	[0.82 - 1.07]	[0.87 - 1.14]
Age			
*18-24 years*	*ref*	*ref*	*ref*
*25-34 years*	1.00 (0.13)	0.90 (0.11)	1.10 (0.14)
	[0.78 - 128]	[0.70 - 1.15]	[0.86 - 1.40]
*35-44 years*	1.03 (0.13)	0.94 (0.12)	1.05 (0.13)
	[0.80 - 1.32]	[0.73 - 1.20]	[0.82 - 1.34]
*45-54 years*	0.91 (0.12)	0.80 (0.10)	0.94 (0.12)
	[0.70 - 1.17]	[0.61 −1.03]	[0.73 - 1.21]
*55-64 years*	0.87 (0.12)	0.76* (0.10)	0.89 (0.12)
	[0.67 - 1.13]	[0.59 - 1.00]	[0.69 - 1.16]
*≥ 65 years*	0.89 (0.12)	0.71** (0.10)	0.78 (0.11)
	[0.68 - 1.17]	[0.54 - 0.93]	[0.59 - 1.01]
Race			
*White*	*ref*	*ref*	*ref*
*Black or African American*	0.66** (0.07)	0.86 (0.10)	0.76** (0.08)
	[0.53 - 0.82]	[0.69 - 1.07]	[0.61 - 0.94]
*Hispanic or Latino*	0.91 (0.10)	0.81* (0.09)	0.98 (0.10)
	[0.74 - 1.12]	[0.66 - 1.00]	[0.79 - 1.20]
*Asian*	0.59** (0.09)	0.84 (0.12)	0.69** (0.10)
	[0.44 - 0.78]	[0.63 - 1.12]	[0.52 - 0.93]
*Other*	0.92 (0.15)	0.80 (0.13)	0.94 (0.16)
	[0.68 - 1.17]	[0.56 - 1.12]	[0.86 - 1.31]
Education			
*High school or less*	*ref*	*ref*	*ref*
*Some college*	0.97 (0.09)	0.94 (0.09)	0.97 (0.09)
	[0.81 - 1.16]	[0.79 - 1.13]	[0.81 - 1.15]
*Tech or associate degree*	0.89 (0.09)	0.96 (0.10)	1.07 (0.11)
	[0.73 - 1.09]	[0.78 - 1.18]	[0.87 - 1.31]
*Bachelor’s degree*	1.09 (0.11)	1.02 (0.11)	1.06 (0.11)
	[0.89 - 1.34]	[0.83 - 1.25]	[0.86 - 1.30]
*Graduate degree*	1.05 (0.15)	1.09 (0.15)	1.13 (0.16)
	[0.80 - 1.34]	[0.83 - 1.43]	[0.86 - 1.48]
Employment Status			
*Employed*	*ref*	*ref*	*ref*
*Unemployed*	0.97 (0.08)	0.97 (0.08)	1.00 (0.08)
	[0.83 - 1.14]	[0.83 - 1.13]	[0.85 - 1.16]
Household Income (US $)			
*< $25*,*000*	*ref*	*ref*	*ref*
*$25*,*000–$49*,*999*	1.21* (0.11)	1.07 (0.10)	1.21* (0.11)
	[1.00 - 1.46]	[0.88 - 1.29]	[1.01 - 1.46]
*$50*,*000–$74*,*999*	1.23* (0.13)	1.24* (0.13)	1.23* (0.13)
	[1.00 - 1.51]	[1.01 - 1.52]	[1.00 - 1.50]
*$75*,*000–$99*,*999*	1.30* (0.17)	1.06 (0.13)	1.31* (0.16)
	[1.02 - 1.67]	[0.83 - 1.35]	[1.02 - 1.67]
*$100*,*000-$149*,*000*	1.45** (0.20)	1.27 (0.17)	1.41** (0.19)
	[1.11 - 1.90]	[0.97 - 1.65]	[1.08 - 1.84]
*$150*,*000 or more*	1.41* (0.24)	1.32 (0.22)	1.35 (0.23)
	[1.01. - 1.96]	[0.95 - 1.83]	[0.97 - 1.87]
Rurality			
*Rural*	*ref*	*ref*	*ref*
*Suburban*	0.99 (0.08)	0.94 (0.08)	0.97 (0.08)
	[0.85 - 1.16]	[0.80 - 1.11]	[0.82 - 1.13]
*Urban*	1.17 (0.11)	0.05 (0.10)	1.08 (0.10)
	[0.97 - 1.41]	[0.88 - 1.26]	[0.90 - 1.29]
Political Identity			
*Democrat*	*ref*	*ref*	*ref*
*Independent*	0.54** (0.05)	0.54** (0.05)	0.58** (0.05)
	[0.45 - 0.64]	[0.46 - 0.64]	[0.49 - 0.69]
*Republican*	0.45** (0.04)	0.45** (0.04)	0.53** (0.05)
	[0.38 - 0.53]	[0.38 - 0.53]	[0.45 - 0.63]
US Census Region			
*Northeast*	*ref*	*ref*	*ref*
*Midwest*	1.00 (0.10)	1.06 (0.11)	1.07 (0.11)
	[0.82 - 1.22]	[0.87 - 1.28]	[0.88 - 1.30]
*West*	1.21 (0.13)	1.26* (0.13)	1.23* (0.13)
	[0.99 - 1.48]	[1.03 - 1.54]	[1.01 - 1.51]
*South*	1.16 (0.11)	1.17 (0.11)	1.14 (0.10)
	[0.97 - 1.39]	[0.98 - 1.40]	[0.95 - 1.36]
Military Status			
*No military experience*	*ref*	*ref*	*ref*
*Military experience*	1.07 (0.11)	1.16 (0.12)	1.07 (0.11)
	[0.87 - 1.30]	[0.94 - 1.42]	[0.88 - 1.31]
Sympathy			
*Unsympathetic*	*ref*	*ref*	*ref*
*Sympathetic*	2.33** (0.16)	2.47** (0.17)	2.28** (0.16)
	[2.03 - 2.68]	[2.15 - 2.83]	[1.98 - 2.61]

Cell entries represent results of ordinal logistic regression models using odds ratios with standard errors in parentheses and confidence intervals in brackets (rounded to two decimal points). From left to right, the dependent variables for the three models indicate that the respondent agrees that (1) immigration enforcement in hospitals will deter undocumented immigrants from seeking needed medical care, (2) immigration enforcement in hospitals will deter undocumented parents of U.S. citizen children from taking their children to get needed medical care, and (3) immigration enforcement in hospitals will deter undocumented immigrants from calling 9-11 in an emergency. Dependent variables for the models are Likert scales ranging from “strongly disagree” (1) to “strongly agree” (5)

## Discussion

Our findings suggest that political identification as a Republican or Independent and being of increased age are consistent predictors of both less awareness of the sensitive locations policy and increased support for rescinding the sensitive locations policy. These findings support the existing literature related to political ideology and attitudes towards immigrants, which consistently demonstrate that those with nationalistic and right-wing authoritarian attitudes are more likely to have negative attitudes towards immigrants [34–38]. Specifically, previous research finds that, while the majority of Americans hold positive views of immigrants, individuals who identify as Republican are more likely to vary in their views of immigrants based on legal status [39]. Republicans tend to hold more negative views of undocumented immigrants, more commonly see immigrants as a threat, and are also more likely to support restrictive immigration policies [40, 41]. Our findings support this existing research and suggest that one’s political party affiliation is a significant indicator of support for more restrictive immigration policies. Our findings add to the literature by suggesting that this support of restrictive immigration policies includes increased support for rescinding the sensitive locations policy.

Our findings also support and add to existing literature that suggests that middle-age and older Americans have less favorable attitudes towards immigrants and that older individuals support more conservative immigration policies [42]. Previous literature on the relationship between age and attitudes towards immigrants is limited and our findings strengthen this literature, pointing to older respondents having less favorable attitudes towards immigrants and more support for rescinding the sensitive locations policy. Given that much of the literature on the relationship between age and attitudes towards immigrants focuses on adults in European countries [42–44], our findings add to the understanding of this relationship in the United States.

Our findings suggest that there are important distinctions based on race regarding beliefs about the impact of rescinding sensitive locations policy. Notably, Hispanic or Latino respondents were more likely to agree that immigration enforcement should not occur in hospitals, schools, and churches compared to White respondents, but Black or African American respondents and Asian respondents were less likely to agree. This racial difference may be because current U.S. immigration enforcement primarily targets Hispanic or Latino individuals. Our findings lend a new perspective to understanding how race and ethnicity influence attitudes towards immigrants and immigration policy. Previous research has indicated that education and political affiliation moderate racial influence [45], but our findings suggest that, with regards to sensitive location policy, race and ethnicity are significant predictors of support regardless of education level or party affiliation.

Our findings also add to the literature related to Black and African American attitudes towards immigrants. Previous research has found that Black and African American individuals tend to hold more liberal immigration views, but the feeling of competing for limited resources with immigrants can lead Black and African American individuals to hold more negative views of immigrants [46]. Additionally, our findings support previous research that shows Asian Americans are the racial group least likely to hold favorable views of undocumented immigrants [47] and that the majority of Asian Americans believe that deporting undocumented immigrants is an important goal [48]. These findings add to the existing understanding of public perceptions related to the impact of immigration policies on health-seeking behavior of undocumented individuals because, to our knowledge, no previous public opinion studies have examined perceptions of the impact of immigration policy on care-seeking among different racial and ethnic groups. Additionally, our findings related to differing perceptions of the impact of immigration policies based on income also provide a new perspective within the existing literature.

Our study demonstrates a relationship between graduate-level education and disagreement with rescinding sensitive location policies. This finding supports previous literature that higher levels of education tend to have more favorable attitudes towards immigrants [41; 45]. As levels of education increase, individuals tend to hold more liberal views regarding immigration policy, though previous research finds this is moderated by party affiliation [45]. Our results support this finding, and show that as one’s education level increases, so does support for maintaining the sensitive locations policy.

Lastly, our findings suggest that individuals in the Western part of the United States are more likely to believe that the rescinding of the sensitive locations policy will deter undocumented individuals from seeking needed medical care. Importantly, geographic location did not determine support for maintaining the sensitive locations policy. As both the West, especially California, and the South, particularly Texas, have large immigrant populations, the lack of significance of geographic location regarding policy action reflects the mixed literature on geography and attitudes towards immigrants [49]. To date, previous research suggests that living in close proximity to immigrant populations increases pro-immigrant attitudes [50, 51], whereas additional literature suggests the opposite [52–55]. Our findings demonstrate that geography has little effect on attitudes towards immigration [56, 57].

Our study has several limitations. First, this study utilizes a cross-sectional study design and was administered during the first days of President Trump’s second administration when many policy changes were underway and partisan tensions were particularly high. Therefore, the cross-sectional design and policy context could make our results prone to bias. Second, it is important to acknowledge that our survey questions were developed before the change to the sensitive locations policy occurred, even as it was administered just after the policy change. As such, several survey questions were sub optimally worded. For example, our survey statement that “I am aware the Immigration and Customs Enforcement (ICE) maintains a policy prohibiting immigration enforcement activities in sensitive locations such as hospitals, schools, and churches” was technically incorrect as the policy had changed by the time the survey was fielded even as it was accurate when our survey was built and received IRB approval. As such, findings from this and other related questions should be assessed with caution.

Next, the study was distributed using an online tool and was only available in English. Therefore, those who lacked English language fluency and access to the internet, were implicitly excluded from the study. Finally, while our survey question asking whether politics related to immigration has ‘no place’ in the US healthcare system was designed to assess public attitudes about immigration enforcement in the healthcare environment, the question was framed overly broadly, and respondents could have simply agreed that politics can influence many aspects of care for immigrants unrelated to the sensitive locations policy, for example related to health access.

Despite differences based on individual characteristics, our findings suggest that the majority of Americans do not think that ICE’s sensitive locations policy should be rescinded and believe that rescinding this policy will deter undocumented immigrants from seeking needed medical care for themselves and their U.S. citizen children. The lack of popular support for the rescinding of sensitive locations policy combined with the known negative health impacts of healthcare avoidance or delaying needed medical care suggests that the sensitive locations policy should be reinstated. Lastly, given the likely physical and mental harm to undocumented individuals and those from mixed status families because of rescinding the policy, steps should be taken by providers to protect undocumented individuals within healthcare settings and policymakers should push to reinstate the sensitive locations policy.

The public health implications of rescinding sensitive locations policy could be substantial if it leads undocumented individuals and their families to delay or avoid needed medical care. Delay or avoidance of medical care can lead to negative health outcomes including uncontrolled chronic disease, infectious disease spread, and premature death. Our findings suggest that Americans have some awareness of the possible public health implications of the policies, though nuances, such as general support for healthcare access, likely exist that the survey did not measure. Generally speaking, however, the majority of Americans did not support enforcement actions in sensitive locations.

In this light, healthcare facilities finding ways to protect undocumented patients and their families seeking out healthcare services is vital to health and would likely be supported by a majority of the US public. Efforts can include ensuring that health facility workers are informed on patient rights regardless of documentation status, designating certain areas of healthcare facilities as private to protect patients unless there is an active warrant, and establishing clear protocols for managing the rescinding of the sensitive locations policy. Analyzing the effectiveness of these potential approaches, and public support for them is an important direction for future research.

Ultimately, our results show that a majority of Americans do not think that ICE’s sensitive locations policy should be rescinded. Given the potential chilling effect this policy change could have on health seeking behavior and the lack of support we identify, there is strong ground to suggest that the sensitive locations policy should be reinstated.

## Supplementary Information

Below is the link to the electronic supplementary material.

Supplementary Material 1

## Data Availability

Data available from corresponding author upon reasonable request.
