# Supplementary material for: U.S. Public Opinion About Immigration Enforcement in Sensitive Locations
Source: J Immigr Minor Health. 2025 Sep 1;28(1):9–22. doi: 10.1007/s10903-025-01772-0 (PMC12882844; doi:10.1007/s10903-025-01772-0)
Supplement: Supplementary file 1 — Supplementary Material 1 [file 10903_2025_1772_MOESM1_ESM.pdf]

National Survey on Immigration Policy and Healthcare Access

Please select your level of agreement with the following statements:

1. All individuals, including those who are undocumented, should be able to see a primary care doctor in the United States.
    a. Strongly agree
    b. Somewhat agree
    c. Neither agree nor disagree
    d. Somewhat disagree
    e. Strongly disagree
2. Only individuals who can pay for healthcare services should be able to see a primary care doctor in the United States.
    a. Strongly agree
    b. Somewhat agree
    c. Neither agree nor disagree
    d. Somewhat disagree
    e. Strongly disagree
3. Only individuals who can pay for healthcare services should be able to see an emergency care doctor in the United States.
    a. Strongly agree
    b. Somewhat agree
    c. Neither agree nor disagree
    d. Somewhat disagree
    e. Strongly disagree
4. U.S. citizens should always be able to get emergency care, even if they do not have insurance.
    a. Strongly agree
    b. Somewhat agree
    c. Neither agree nor disagree
    d. Somewhat disagree
    e. Strongly disagree
5. Undocumented individuals should always be able to get emergency care, even if they do not have insurance.
    a. Strongly agree
    b. Somewhat agree
    c. Neither agree nor disagree
    d. Somewhat disagree
    e. Strongly disagree
6. All individuals who call 9-11 should be transported to a hospital if needed, regardless of their ability to pay.

    a. Strongly agree
    b. Somewhat agree
    c. Neither agree nor disagree
    d. Somewhat disagree
    e. Strongly disagree
7. All individuals who call 9-11 should be transported to the hospital if needed, regardless of their immigration status.
    a. Strongly agree
    b. Somewhat agree
    c. Neither agree nor disagree
    d. Somewhat disagree
    e. Strongly disagree
8. Undocumented immigrants get free healthcare.
    a. Strongly agree
    b. Somewhat agree
    c. Neither agree nor disagree
    d. Somewhat disagree
    e. Strongly disagree
9. It is easier for undocumented immigrants get healthcare than it is for U.S. citizens without insurance.
    a. Strongly agree
    b. Somewhat agree
    c. Neither agree nor disagree
    d. Somewhat disagree
    e. Strongly disagree
10. Being able to see a doctor is important to living a healthy life.
    a. Strongly agree
    b. Somewhat agree
    c. Neither agree nor disagree
    d. Somewhat disagree
    e. Strongly disagree
11. Healthcare is a basic human right.
    a. Strongly agree
    b. Somewhat agree
    c. Neither agree nor disagree
    d. Somewhat disagree
    e. Strongly disagree
12. Politics related to immigration have no place in the U.S. healthcare system.
    a. Strongly agree
    b. Somewhat agree
    c. Neither agree nor disagree

    d. Somewhat disagree
    e. Strongly disagree
13. I was aware that Immigration and Customs Enforcement (ICE) maintains a policy prohibiting immigration enforcement activities in sensitive locations such as hospitals, schools, and churches.
    a. Strongly agree
    b. Somewhat agree
    c. Neither agree nor disagree
    d. Somewhat disagree
    e. Strongly disagree
14. Immigration enforcement should not occur in hospitals.
    a. Strongly agree
    b. Somewhat agree
    c. Neither agree nor disagree
    d. Somewhat disagree
    e. Strongly disagree
15. Immigration enforcement should not occur in schools.
    a. Strongly agree
    b. Somewhat agree
    c. Neither agree nor disagree
    d. Somewhat disagree
    e. Strongly disagree
16. Immigration enforcement should not occur in churches.
    a. Strongly agree
    b. Somewhat agree
    c. Neither agree nor disagree
    d. Somewhat disagree
    e. Strongly disagree
17. Immigration enforcement in hospitals will deter undocumented immigrants from seeking needed healthcare.
    a. Strongly agree
    b. Somewhat agree
    c. Neither agree nor disagree
    d. Somewhat disagree
    e. Strongly disagree
18. Immigration enforcement in hospitals will prevent U.S. citizen children (0-18 years of age) with undocumented parents from seeking needed healthcare.
    a. Strongly agree
    b. Somewhat agree
    c. Neither agree nor disagree
    d. Somewhat disagree

    e. Strongly disagree
19. Which state do you live in? [drop down menu]
20. How do you describe yourself (select one):
    a. Male
    b. Female
    c. Trans Male/Trans Man
    d. Trans Female/Trans Woman
    e. Genderqueer/Gender Non-conforming
    f. Other
21. How old are you?
    a. 18-24 years old
    b. 25-34 years old
    c. 35-44 years old
    d. 45-54 years old
    e. 55-64 years old
    f. 65-74 years old
    g. 75+
22. Which race best describes you?
    a. American Indian or Alaska Native
    b. Asian
    c. Black or African American
    d. Native Hawaiian or Pacific Islander
    e. White
    f. Hispanic or Latino
    g. Two or more races
    h. Other
23. What is your annual household income?
    a. Less than $10,000
    b. $10,000 to $24,999
    c. $25,000 to $49,999
    d. $50,000 to $74,999
    e. $75,000 to $99,999
    f. $100,000 to $149,999
    g. $150,000 or more
24. What is the highest level of education you have completed or the highest degree you have received?
    a. Less than high school
    b. High school diploma or equivalent
    c. Some college, but no degree
    d. Technical certification/degree
    e. Associate’s degree

    f. Bachelor’s degree
    g. Master’s degree
    h. Doctoral or professional degree
25. Which best reflects your employment status?
    a. Employed, full-time
    b. Employed, part-time
    c. Unemployed, looking for work
    d. Unemployed, not looking for work
    e. Retired
    f. Stay-at-home caregiver for family member
    g. Full-time student
26. What type of area do you live in?
    a. Rural
    b. Suburban
    c. Urban
27. Are you a U.S. military veteran?
    a. Yes
    b. No
28. Which best describes your health insurance status?
    a. Insured through my employer (non-military)
    b. Insured through a marketplace or private option
    c. Insured through a public option (i.e., Medicaid, Medicare)
    d. Insured through the U.S. military (i.e., Veterans Affairs)
    e. Currently uninsured
29. At any point in your adult life, have you been uninsured?
    a. Yes
    b. No
30. Which political affiliation do you most closely identify with:
    a. Democrat
    b. Independent
    c. Republican
31. In general, how sympathetic would you say that you are towards immigrants who are in the United States illegally?
    a. Very unsympathetic
    b. Somewhat unsympathetic
    c. Somewhat sympathetic
    d. Very sympathetic
